# New records of *Hepatozoon* and *Oswaldofilaria* from saltwater crocodiles (*Crocodylus porosus*) in Australia

**DOI:** 10.1016/j.ijppaw.2024.100926

**Published:** 2024-03-18

**Authors:** T. Franciscus Scheelings, Anson V. Koehler, Robin B. Gasser

**Affiliations:** Melbourne Veterinary School, Faculty of Science, The University of Melbourne, Parkville, Victoria, 3010, Australia

**Keywords:** Crocodylus porosus, Saltwater crocodile, Haemoparasites, Hepatozoon, Oswaldofilaria, Australia

## Abstract

Diseases affecting wild Australian saltwater crocodiles (*Crocodylus porosus*) are rarely reported due to the difficulty in capturing animals and obtaining samples. In this investigation, we identified two haemoparasites (*Hepatozoon* and a filarial nematode) in saltwater crocodiles in Darwin, Australia. Light microscopic examination identified *Hepatozoon* in 7/7 (100%) wild crocodiles and in 2/20 (10%) of captive ones. When genomic DNAs from these same samples were further investigated using polymerase chain reaction (PCR)-based sequencing, we detected *Hepatozoon* in all 27 blood samples. Using both microscopy and PCR-based sequencing, we detected a filarial worm (proposed to be *Oswaldofilaria*) in one of 20 captive crocodiles. The sequence data were compared with sequence data available in public databases, and phylogenetic analyses indicated that the operational taxonomic units of *Hepatozoon* and *Oswaldofilaria* discovered here in these crocodiles are likely new species. This study is the first to use molecular tools to explore haemoparasites in Australian saltwater crocodiles and highlights the importance of health investigations in poorly studied vertebrate hosts.

## Highlights

• Both wild and captive crocodiles were found to be infected with a *Hepatozoon* sp.
• A single captive crocodile was found to infected with an *Oswaldofilaria* sp.
• Molecular analysis of parasites suggests that they are likely to be novel species.
• Pathogenicity, and lifecycle of these parasites in crocodiles are still unknown.

## Graphical abstract

**Figure:**
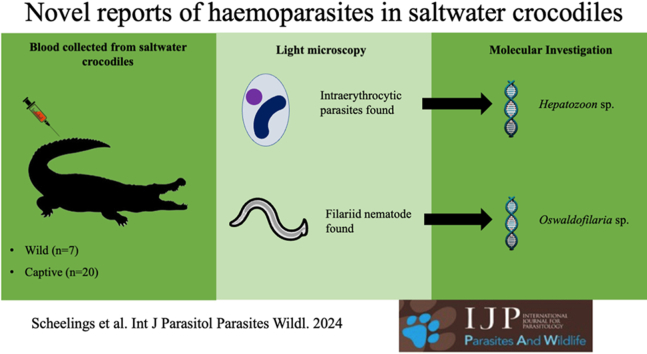

## 1 Introduction

The saltwater crocodile (*Crocodylus porosus*) is the largest extant species of crocodilian, with individuals capable of obtaining lengths exceeding 6 m and a mass of >1000 kg (Webb et al., 2010; Britton et al., 2012). This species has a broad geographic distribution, with a range extending from southern India to Sri Lanka, throughout southeast Asia, east through the Philippines, and south through Indonesia, Papua New Guinea, the Solomon Islands and the north of Australia (Webb et al., 2010). In Australia, saltwater crocodiles were almost hunted to extinction in the first half of the 20th century; however, their populations have recovered to pre-European settlement levels following their protection in 1971 (Fukuda et al., 2011). Saltwater crocodiles are aggressive predators, and as such, there are relatively few proactive studies of the health and diseases of wild populations in comparison to other Australian fauna. As apex predators, crocodilians are an important ecological and evolutionary group of animals (Lourenco-de-Moraes et al., 2023). Despite the practical difficulties in conducting research on them, understanding how infectious diseases may play a role in population dynamics is imperative to their conservation and warrants greater scientific investment.

Haemoparasitaemia is a common finding in free-ranging reptiles and infections have been reported in all taxa with varying frequencies (Peirce and Adlard, 2004; Telford, 2009; Vilcins et al., 2009; Herbert et al., 2010; Scheelings and Rafferty, 2012; Scheelings et al., 2016; Ungari et al., 2022). However, one group that is poorly represented in the literature is the Crocodilia, with only scant accounts of haemoparasitism occurring in this Order. Hemogregarines have been observed in crocodilians, with *Hemogregarina crocodilinorum* commonly found in the blood of wild American Alligators (*Alligator*) (Walden et al., 2021). Undescribed species of *Hemogregarina* have been reported in freshwater crocodiles (*Crocodylus noveaguineae*) and saltwater crocodiles (Ladds et al., 1995) in New Guinea. Additionally, *Hepatozoon* spp. Have been observed in the peripheral blood of freshwater crocodiles (*Crocodylus johnstoni*) (Scheelings et al., 2016) and African dwarf crocodiles (*Osteolaemus tetraspis*) (Huchzermeyer and Agnagna, 1994). Despite previous reports of the genus *Hemogregarina* occurring in crocodilians, all haemogregarines infecting these species have now been assigned to the genus *Hepatozoon* (Smith, 1996).

A second important group of haemoparasites found in crocodilians are nematodes of the superfamily Filarioidea (order Spirurida), in particular the genus *Oswaldofilaria* (Walden et al., 2021). *Oswaldofilaria kanbaya* has been reported in saltwater crocodiles (Manzanell, 1986), *O. medemi* in smooth-fronted caimans (*Paleosuchus trigonatus*) (Marinkelle, 1981), *O. bacillaris* from spectacled caimans (*Caiman crocodilus*) and black caimans (*Melanosuchus niger*) (Travassos, 1933), and *O. versterae* from Nile crocodiles (*Crocodylus niloticus*) (Bain et al., 1982). In each of these cases, parasites were present in the peripheral blood as microfilariae that had been produced by adult nematodes encysted elsewhere in the body.

At present, all descriptions of haemoparasites in crocodilians are based on morphology alone, which is problematic due to the disagreement among some parasitologists on the importance of specific morphological characteristics, especially for apicomplexan parasites (Barta et al., 2012). In this investigation, we used classical light microscopy and molecular-phylogenetic approaches to characterise two haemoparasites from saltwater crocodiles in Darwin, Australia.

## 2 Materials and methods

### 2.1 Ethics statement

This study was approved by The University of Melbourne Office of Research Ethics and Integrity (Ethics ID: 2022-24808-32226-4), and all experiments were performed in accordance with relevant guidelines and regulations. Crocodiles were trapped under the permit 72249 from the Parks and Wildlife Commission of the Northern Territory, Australia.

### 2.2 Source of animals

Free-ranging saltwater crocodiles (n = 7) were trapped in various locations within the Darwin Harbour area, as part of the Northern Territory's Problem Crocodile Management Program (Fukuda et al., 2014) in April 2023. Crocodiles were captured in marine waters using large aluminium traps suspended in the water with flotation devices and baited with sections of wild pig. Traps were checked three times per week, depending on local tide and ocean conditions. When a crocodile was found within a trap, it was removed using ropes attached to the jaws and then restrained by taping the mouth shut and covering the eyes with gaffer tape. Mobility was further restricted by securing the hind limbs with ropes. The crocodile was then transported to a central processing hub for sampling. Following sample collection, the crocodile was collected by a private enterprise for commercial use. Captive crocodiles (n = 20) were all sampled at a privately-owned zoological institution in Darwin. Crocodiles were housed in a large freshwater enclosure and were manually restrained using ropes and tape in a similar manner to the wild animals. After sampling, all captive crocodiles were permitted to return to their enclosures. Following restraint, 10 ml of blood was collected from the ventral coccygeal vein via a lateral approach using a 10 ml syringe attached to an 18-gauge needle.

### 2.3 Preparation of samples and blood smears

Immediately after collection, 250 μL of blood were transferred into a lithium heparin container (Sarstedt AG & Co., Nümbrecht, Germany) and the remainder into a gel clot activator tube (Sarstedt AG & Co., Nümbrecht, Germany) for blood coagulation. Blood tubes were then placed into a portable ice pack and taken to the laboratory for processing. In the laboratory, blood smears were made by placing a drop (50 μl) of uncoagulated blood on to a microscope slide and smearing it with a cover slip (Campbell and Grant, 2022). Slides were stained with Wright's Giemsa using an automatic stainer (Siemens Hematek) (Siemens Australia, Bayswater, Australia). Parasitaemia was determined by examining blood smears using a light microscope (Leica, Wetzlar, Germany) at 100-times magnification and by counting the number of parasites in 1000 erythrocytes. Blood in the plain tube was centrifuged, the serum was removed, and the remaining red blood cell (RBC) pellet was stored at −80 °C until molecular analysis was undertaken.

### 2.4 Molecular analysis

Genomic DNA was extracted from ∼200 μl of the RBC pellet by digestion with proteinase K (10 μg/μl) (Promega, USA) for 5 h at 56 °C for (using a Thermomixer at 650 rpm) and then purified using the DNeasy PowerSoil Pro Kit (Qiagen, Netherlands) following the manufacturer's protocol.

PCR was used to amplify distinct gene regions specifically from apicomplexans (one-step PCR) and filarioid nematodes (two-step, nested PCR) from the purified genomic DNA sample/s. Each PCR was performed in a volume of 50 μl containing Go*Taq* Flexi buffer (Promega, USA), 3.0 mM of MgCl_2_, 200 μM of each dNTP, 50 pmol of each primer, 1 U of Go*Taq* DNA polymerase (Promega, USA) and 2 μl of genomic DNA (for single-step PCR) or 1 μl of primary amplicon (for nested PCR). Known test-positive, test-negative and no-template controls were included in each PCR run.

For apicomplexans, part of the small subunit of nuclear RNA (SSU; 889 bp) was amplified using the primers HAM-F (forward: 5′- GCCAGTAGTCATATGCTTGTC-′3) and HepR900 (reverse: 5′- CAAATCTAAGAATTTCACCTCTGAC-′3) (Ujvari et al., 2004; Criado-Fornelio et al., 2006; Netherlands et al., 2018). PCR cycling conditions were: an initial denaturation at 94 °C for 5 min, followed by 35 cycles of denaturation at 94 °C for 30 s, annealing at 61 °C for 30 s, and extension at 72 °C for 120 s, with a final extension step at 72 °C for 5 min.

For filarioid nematodes, part of the mitochondrial cytochrome *c* oxidase subunit 1 gene (*cox*1; 681 bp) was amplified by nested PCR. In the primary PCR (to amplify a product expected to be ∼970 bp), primers FCo1extdF1 (forward: 5′- TAT AAT TCT GTT YTD ACT A -′3) and FCo1extdR1 (reverse: 5′- ATG AAA ATG AGC YAC WAC ATA A -′3) (Lefoulon et al., 2015) were used for amplification from 2 μl of the genomic DNA sample. In the second PCR, primers COIintF (forward: 5′- TGA TTG GTG GTT TTG GTA A -′3) and COIintR (reverse: 5′- ATA AGT ACG AGT ATC AAT ATC -′3) (Casiraghi et al., 2004) were used for amplification from 1 μl of primary amplicon. For both PCR steps, the cycling conditions were the same: an initial denaturation at 94 °C for 5 min, followed by 35 cycles of denaturation at 94 °C for 30 s, annealing at 52 °C for 45 s, and extension at 72 °C for 60 s, with a final extension step at 72 °C for 5 min (Lefoulon et al., 2015).

Following PCR, all amplicons were subjected to conventional agarose gel electrophoresis (Koehler et al., 2016) to examine the sizes and intensity of the products. PCR products that represented single bands of the appropriate size (889 bp or 681 bp) were treated with the enzymes *Exo* I (Thermofisher, USA) and a thermosensitive alkaline phosphatase (FastAP, Thermofisher, USA) to remove primers and then subjected to bi-directional (Sanger) sequencing using a standard protocol (Koehler et al., 2016).

### 2.5 Phylogenetic analysis

Sequence data obtained were examined for quality and deposited in the National Center for Biotechnology Information (NCBI) database under GenBank accession nos. PP112021 and PP117936). The sequences obtained were compared with respective sequences in the GenBank database using the Basic Local Alignment Search Tool (BLAST; www.ncbi.nlm.nih.gov), and then aligned with suitable reference sequences and selected outgroup taxa represented in this database. Sequences were compared in a pairwise manner, and sequence identities recorded using Geneious Prime v. 2023.2.1 software (www.geneious.com). Sequences were aligned using the program Muscle (Edgar, 2004), and alignments adjusted manually using the program Mesquite v.3.61 (Maddison and Maddison, 2017).

Phylogenetic analysis of sequence data was conducted using the neighbour-joining (NJ) distance method (Saitou and Nei, 1987) in the program MEGA v.11.0.11 (Tamura et al., 2021). Evolutionary distances were computed using the ‘number of differences’ method (Nei and Kumar, 2000), including ‘transitions and transversions’ for the nucleotide data. Rates of evolution among sites were considered uniform and gaps were treated using pairwise deletion. A total of 2000 bootstrap replicates were performed and are reported as bootstrap support percentages (bs). In the analyses, two *Dactylosoma* species were employed as outgroups for *Hepatozoon* species, while two *Spirocerca* species were used as outgroups for filarioid nematodes.

## 3 Results and discussion

### 3.1 Characterisation of an apicomplexan reveals a new record of hepatozoon

**Fig. 1:**
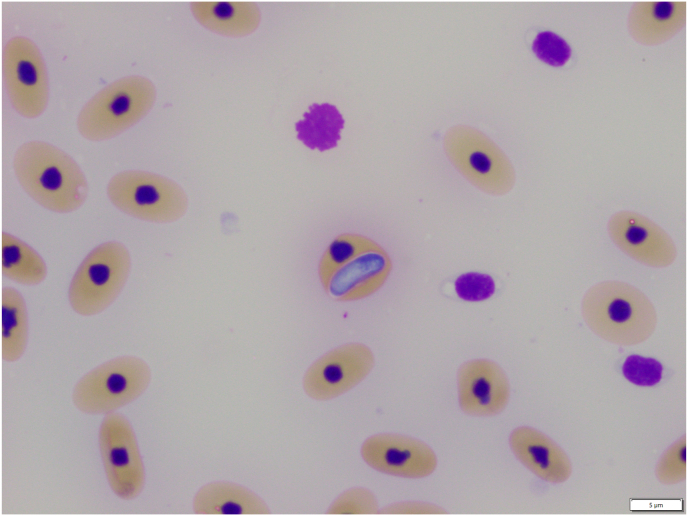
A stage of *Hepatozoon* identified in erythrocytes in blood smears from a saltwater crocodile (*Crocodylus porosus*). Stained with Wright's Giemsa; examined at 100× magnification; scale bar = 5 μm.

Haemogregarines were detected both by light microscopic examination of blood smears and by molecular methods in free-ranging and captive saltwater crocodiles (Fig. 1). Light microscopic examination of blood smears revealed that 7/7 (100%) of wild crocodiles, and 2/20 (10%) captive crocodiles were infected with an intra-erythrocytic haemogregarine parasite. For infected crocodiles, parasites were observed at a rate of 1–5 parasites per 1000 R BCs. Parasites were sausage-shaped with a light blue cytoplasm and a central, dark-staining nucleus. The presence of haemogregarines within erythrocytes resulted in occasional displacement of the nucleus but no obvious damage to the cell.

**Fig. 2:**
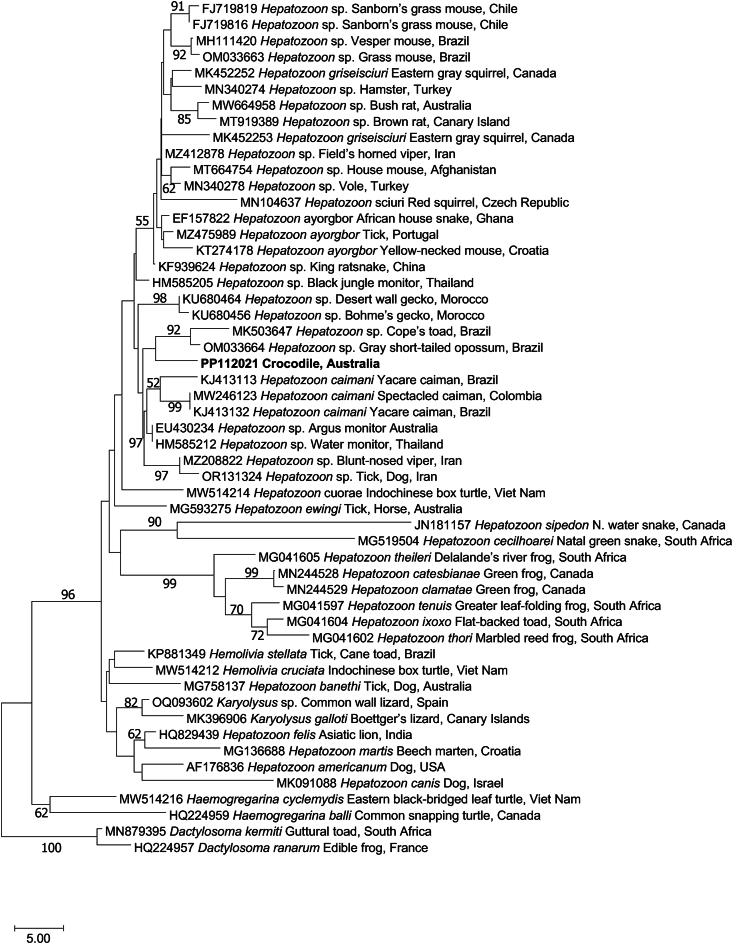
Relationship of a new species of *Hepatozoon* (bold) identified in erythrocytes from the blood of the saltwater crocodile with representative taxa represented in the GenBank database, established based on a phylogenetic analysis of sequence data from part of the small subunit of nuclear ribosomal RNA gene (*SSU;* 889 bp) employing the neighbour-joining distance method. Branch supports are represented by neighbour-joining bootstrap percentages. Species of *Dactylosoma* were used as outgroups.

All crocodiles were PCR test-positive for apicomplexan DNA. Six amplicons from both the wild and captive saltwater crocodiles underwent sequencing and identical apicomplexan sequences were obtained (GenBank accession no. PP112021). This sequence has the highest identity (870/889 bp, 98%) to another sequence with GenBank accession no. EF157822 representing *Hepatozoon ayorgbor* from an African house snake (*Lamprophis fuliginosus*, Ghana, Africa). Phylogenetic analysis positions our unique apicomplexan sequence (PP112021) amongst members of the genus *Hepatozoon* from amphibians, reptiles and mammals, and in the vicinity of a sequence from *Hepatozoon caimani* - the sole representative *Hepatozoon* from a crocodilian in GenBank (Fig. 2).

According to Duszynski et al. (2020), there are four recognised species of *Hepatozoon* in crocodilians: *Hepatozoon caimani* (South American caimans), *Hepatozoon crocodilinorum* (American alligator), *Hepatozoon pettiti* and *Hepatozoon sheppardi* (both from Nile crocodiles). Although intraerythrocytic parasites have been reported in both salt and freshwater crocodiles (Owens et al., 1988), their clincial significance remains unknown. Furthermore, it remains uncertain whether these previously-reported parasites were hepatozoons.

Although we identified and characterised *Hepatozoon* parasites, we did not identify any ectoparasites on the crocodiles that might serve as vectors. In other aquatic reptiles, such as chelonians, leeches have been identified as important transmitters of haemoparasite infections, even between phylogenetically distinct hosts (Maricic et al., 2023; Verneau et al., 2023). Leeches are believed to be the host for *H. crocodilinorum* (Khan et al., 1980). Leech infestations have been reported in saltwater crocodiles, but not in animals originating in Australia (Watermolen, 2021). However, leeches are known to parasitise freshwater crocodiles in Australia (Webb and Manolis, 1983), which are sympatric with saltwater crocodiles across parts of their range, including the Northern Territory (Cogger, 2014). An interesting aspect of the epidemiology of the *Hepatozoon* infections in this study is that crocodiles were sourced from both freshwater and saltwater environments, as it appears that leeches do not infect crocodilians residing in saline water (Webb and Messel, 1977; Montague, 1984). However, this does not rule them out as a potential vector, as saltwater crocodiles are highly mobile and will readily move between freshwater and marine environments (Webb et al., 2010). Thus, it is possible that wild crocodiles became infected with leeches while inhabiting freshwater sections of rivers or inland swamps and lakes, and that the leeches were shed as the crocodiles transitioned into tidal areas. Alternatively, other vectors, such as biting arthropods, might be responsible for transmitting the parasites, as observed for tsetse flies and *H. pettiti* (Hoare, 1932), or mosquitoes and *H. caimani* (Pessôa et al., 1972). Future studies of apicomplexan parasites in Australian crocodiles should focus on their biology and the vectors responsible for their transmission.

### 3.2 Characterisation of a filarioid reveals a new record of oswaldofilaria

**Fig. 3:**
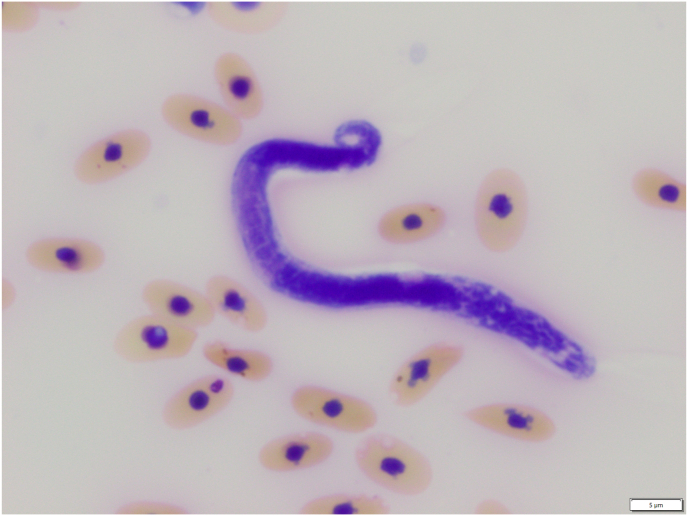
Microfilaria of a species of *Oswaldofilaria* in a blood smear from a saltwater crocodile (*Crocodylus porosus*). Stained with Wright's Giemsa; examined at 100-times magnification; scale bar = 5 μm.

**Fig. 4:**
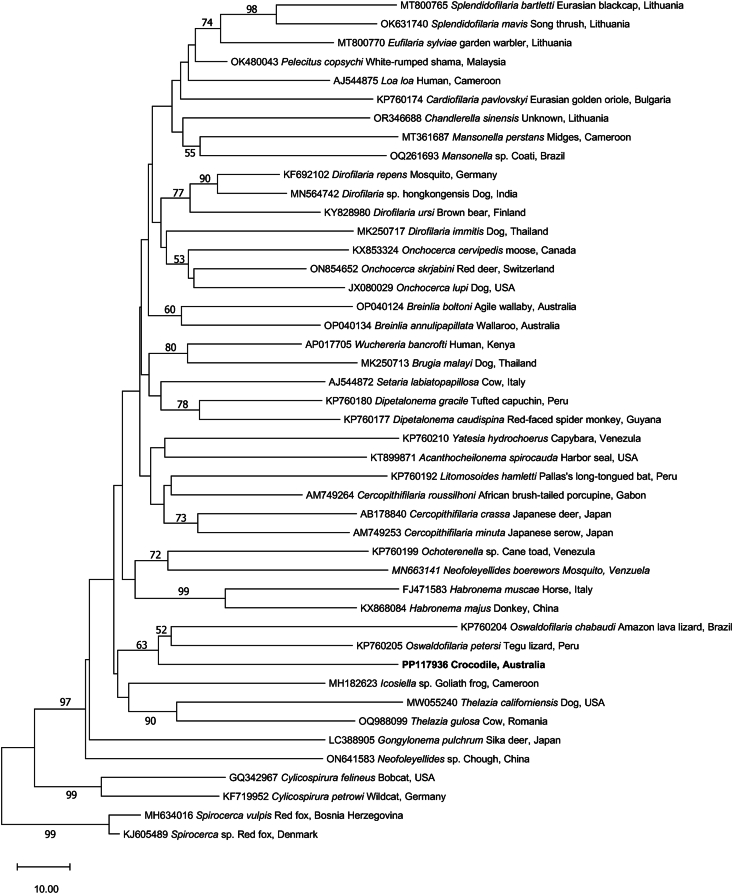
Relationship of a new species of *Oswaldofilaria* (bold) from the blood of the saltwater crocodile with representative taxa represented in the GenBank database, established based on a phylogenetic analysis of sequence data from part of the mitochondrial cytochrome *c* oxidase subunit 1 gene (*cox*1; 681 bp) employing the neighbour-joining distance method. Branch supports are represented by neighbour-joining bootstrap percentages. Members of the genus *Spirocerca* were used as outgroups.

A microfilaria (Fig. 3) was detected by microscopy in the blood of one of the captive saltwater crocodiles examined. This result concurred with nested PCR results in that one *cox*1 amplicon was obtained following PCR of X blood genomic samples from the same crocodiles. The cox1 sequence obtained from this amplicon had 87% (592/681 bp) identity with the sequence with GenBank accession no. KF692102 representing *Dirofilaria repens* from a mosquito in Germany. Phylogenetic analysis positions this novel filarioid nematode amongst Oswaldofilarid species from reptiles (Fig. 4). In reptiles, Oswaldofilarinae, a subfamily within the Onchocercidae, are key nematodes that parasitises crocodilians, and adult nematodes are found in extraintestinal sites (Walden et al., 2021). Microfilariae are released into the circulatory system, and bloodsucking arthropods (e.g., ticks or mosquitoes) become infected when they take a blood meal and then transmit them further (Walden et al., 2021). Filarioid infections in reptiles appear to be rarely associated with clinical disease, and most infections are incidental findings during necropsy examination of carcasses (Walden et al., 2021). In our investigation, no post-mortem examinations were conducted on any animal, such that no definitive statement can be made about tissue predilection of adult nematodes or whether they were associated with any pathological changes in the crocodiles. The nematode characterised here may be *O. kanbaya* (Manzanell, 1986), but further comparative studies of adult worms from saltwater crocodiles would be necessary to test this hypothesis.

## 4 Conclusions

Here, we discovered two new records of haemoparasites in saltwater crocodiles in Australia – one a species of *Hepatozoon* and the other a species of *Oswaldofilaria*. Although infection with these genera of parasites is not known to be associated with clinical disease in reptiles, no attempt has yet been made to assess their effects on host physiology, such that it is unknown whether they are pathogenic or not. An improved understanding of host-parasite relationships is important, because, under particular conditions, non-pathogenic parasites can induce disease and become a cause of concern for population health. Therefore, future work should focus on the epidemiology and pathogenicity of haemogregarines and filarioid nematodes in wild crocodilians.

## Declarations of competing interests

The authors declare no conflict of interest.

## CRediT authorship contribution statement

**T. Franciscus Scheelings:** Conceptualization, Data curation, Formal analysis, Investigation, Methodology, Writing – original draft, Writing – review & editing. **Anson V. Koehler:** Conceptualization, Data curation, Formal analysis, Investigation, Methodology, Writing – original draft, Writing – review & editing. **Robin B. Gasser:** Conceptualization, Data curation, Formal analysis, Methodology, Writing – original draft, Writing – review & editing.
